# Comparison of adverse effects of anti-tumor therapy for breast cancer shortly after COVID-19 diagnosis vs. the control period

**DOI:** 10.3389/fonc.2023.1203119

**Published:** 2023-08-21

**Authors:** Mao Ding, Hongyu Xiang, Jingming Ye, Yuanjia Cheng, Qian Liu, Ling Xu

**Affiliations:** Department of Thyroid and Breast Surgery, Peking University First Hospital, Beijing, China

**Keywords:** breast cancer, chemotherapy, targeted therapy, COVID-19, adverse events

## Abstract

**Background:**

COVID-19 is an acute infectious disease caused by SARS-CoV-2. The best time to restart antitumor therapy in breast cancer patients after SARS-CoV-2 infection is unknown. This study aimed to evaluate treatment-related adverse events in breast cancer patients who received antitumor therapies within a short time after SARS-CoV-2 infection (observation) as well as before (control) and to provide safety data.

**Methods:**

We conducted a self-controlled cohort study using the data from the Breast Disease Center of Peking University First Hospital. We identified patients who received antitumor therapy within 28 days after COVID-19 infection between December 20, 2022, and January 20, 2023. The primary outcome was treatment-related adverse events. McNemar’s test was used to compare the incidence rate of adverse reactions between periods.

**Results:**

We identified 183 patients with breast cancer, of whom 109 were infected with SARS-CoV-2 within 28 days before antitumor treatment and were included. In total, 28 patients (25.7%) received neoadjuvant therapy, 60 (55.0%) received adjuvant therapy, and 21 (19.3%) received advanced rescue therapy. None of patients required hospitalization for severe or critical COVID-19, but 15 patients (13.8%) still had sequelae of COVID-19 while receiving antitumor treatment. The most common adverse events were peripheral neuropathy (n = 32 [29.4%]), pain (n = 29 [26.6%]), fatigue (n = 28 [25.7%]), nausea (n = 23 [21.1%]), and neutropenia (n = 19 [17.4%]). There was no increased risk of overall treatment-related adverse events (n = 87 [79.8%] vs. n = 91 [83.5%]; p = 0.42) or serious adverse events (n = 13 [11.9%] vs. n = 12 [11.0%]; p = 1.00) from receiving antitumor therapy shortly after the diagnosis of COVID-19. We also found no increased risk in subgroup analyses, and no patients discontinued antitumor therapy due to adverse events.

**Conclusion:**

Restarting antitumor therapy 2-4 weeks after having mild or moderate COVID-19 is a relatively safe strategy for breast cancer patients that does not increase the risk of treatment-related adverse events.

## 1 Introduction

Coronavirus disease 2019 (COVID-19) is an acute infectious disease caused by severe acute respiratory syndrome coronavirus 2 (SARS-CoV-2). There are significant individual differences in clinical severity, with the majority of patients being asymptomatic and mildly ill; however, a minority of infected patients become critically ill or die (1). Studies have shown that compared with the general population, cancer patients are at a higher risk of contracting COVID-19 due to their weakened immune function and have higher rates of severe COVID-19 and COVID-19-related mortality (2, 3). In the early stage of the outbreak, the diagnosis and treatment of patients were delayed to varying degrees. With the increase in the cumulative number of people affected by the pandemic, SARS-CoV-2 infection while receiving tumor treatment will become a common phenomenon, and thus, the optimal timing of restarting antitumor therapy will become a new challenge. Existing research focuses on the impact of chemotherapy, molecular targeted therapy, immunotherapy, etc., on the risk of severe COVID-19 and COVID-19-related death (4–6). However, there is still controversy regarding the adverse reactions related to tumor treatment after infection in patients with solid tumors, and there are few relevant reports that include data for the Omicron variant (7, 8).

Breast cancer is the most common malignant tumor in women (9). With the establishment of molecular typing–based diagnosis and treatment models, a comprehensive treatment system that includes chemotherapy, targeted therapy, endocrine therapy, and immunotherapy has been developed. Compared with other solid tumors, evidence of the safety of post-COVID-19 tumor treatment is urgently needed. In the past, the number of patients infected with COVID-19 in China was relatively small. Since the Comprehensive Team of the State Council’s Joint Prevention and Control Mechanism in Response to the Novel Coronavirus Pneumonia Outbreak issued the “Notice on Further Optimizing the Implementation of COVID Prevention and Control Measures” on December 7, 2022, epidemic prevention and control policies have been adjusted. As increasingly more breast cancer patients are infected with SARS-CoV-2, data on post-infection adverse reactions in patients undergoing systemic treatment for breast cancer have gradually accumulated.

The aim of this article is to evaluate treatment-related adverse reactions in breast cancer patients treated at the Breast Disease Center of Peking University First Hospital who received antitumor therapy within a short period of time after SARS-CoV-2 infection and to provide safety data for restarting antitumor therapy after SARS-CoV-2 infection.

## 2 Methods

### 2.1 Study population

**Figure 1:**
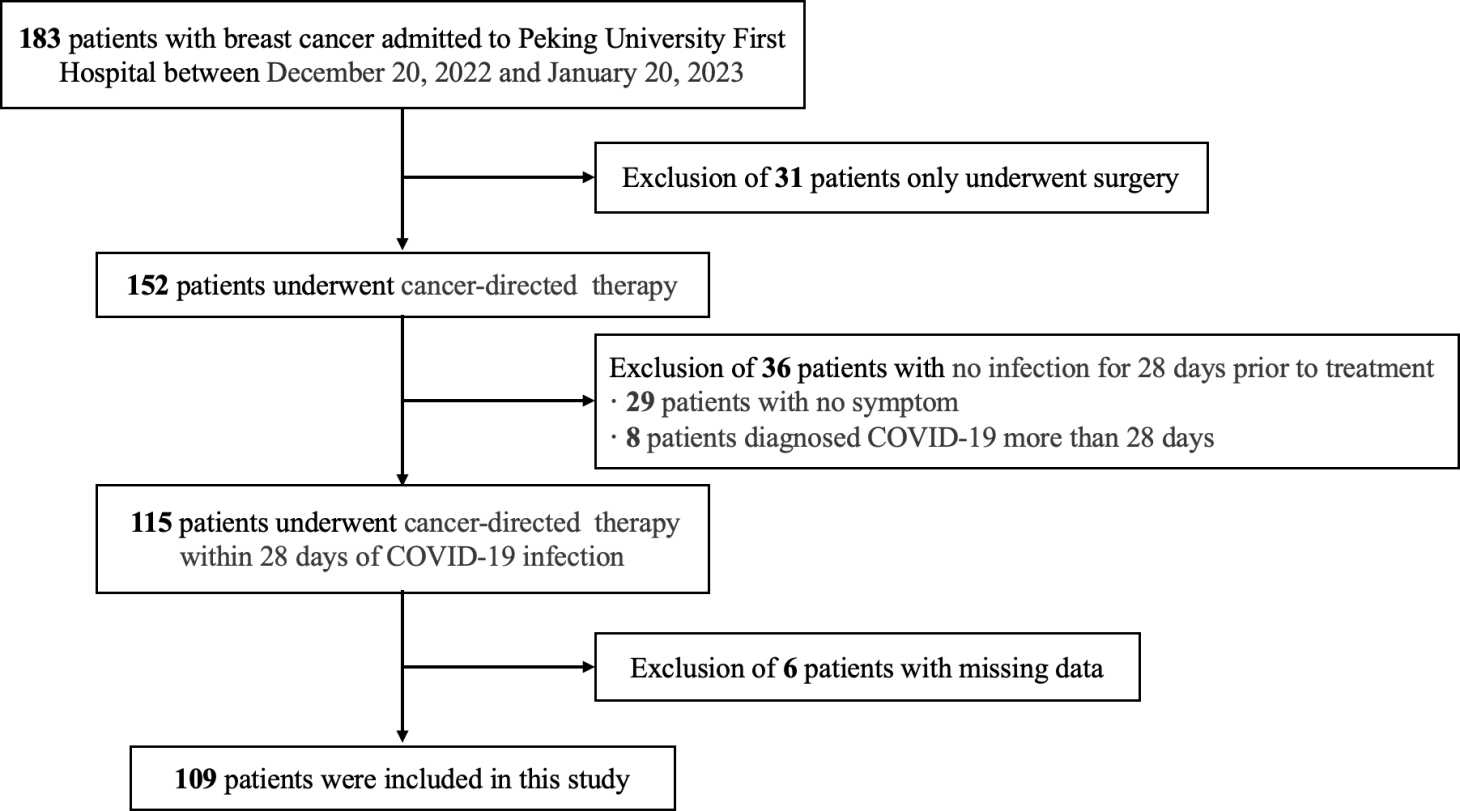
Flow chart of the inclusion of the patients.

Patients who were hospitalized for breast cancer treatment at the Breast Disease Center of Peking University First Hospital between December 20, 2022, and January 20, 2023, were included in this retrospective study. The inclusion criteria were as follows: (a) received at least one systemic therapy for breast cancer, including but not limited to chemotherapy, targeted therapy, endocrine therapy, bone-modifying therapy, and immunotherapy; (b) diagnosed with COVID-19 within 28 days before the current treatment; and (c) had complete follow-up data of treatment-related adverse reactions. The exclusion criteria were as follows: (a) only received surgical treatment; (b) not infected with SARS-CoV-2, or the interval between the first antitumor treatment after infection and SARS-CoV-2 infection was greater than 28 days; and (c) data on treatment-related adverse reactions were lacking and could not be retrospectively collected (**Figure 1**). Clinicopathological data of breast cancer patients were obtained from medical records, including age, sex, body mass index (BMI), history of comorbidities (including diabetes, hypertension, heart, lung, liver, and kidney disease, or other malignancy) and tumor pathology. Staging was conducted using American Joint Committee on Cancer (AJCC) 8th edition (10).

### 2.2 Study design

**Figure 2:**
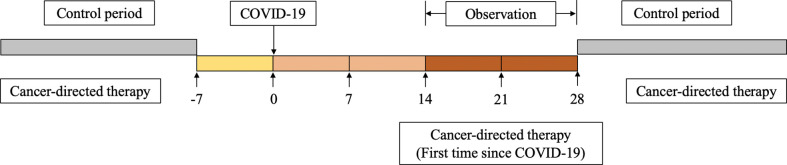
Overview of self-controlled case series study design, including treatment period, period before exposure to SARS-CoV-2, and control period.

This was a retrospective self-controlled trial to determine the difference in treatment-related adverse events in patients who received both antitumor therapy within 28 days after COVID-19 infection and antitumor therapy in the control period. The patients received the same antitumor therapy during the observation and control period, and the dose of drugs was also not adjusted. In this way, the influence of confounding factors such as age, body mass index (BMI), comorbidities, treatment plan and tumor stage were reduced. Considering that treatment-related adverse reactions and COVID-19 may have similar clinical manifestations, the 7 days before infection were not used as a control period (**Figure 2**). For most patients, the last antitumor therapy before COVID-19 was chosen as the control, except for those who were first diagnosed with breast cancer during the pandemic. To ensure the safety of patients and reduce the risk of severe COVID-19 and COVID-19-related death, breast cancer patients were considered candidates to restart antitumor treatment no sooner than 14 days after the diagnosis of COVID-19 and had at least one negative SARS-CoV-2 nucleic acid test result, in accordance with the National Comprehensive Cancer Network (NCCN) “Prevention and Treatment of Cancer-Related Infections (Version 3.2022)” guidelines (11) and the “Chinese Expert Consensus on Issues Related to the Protection, Diagnosis and Management of Solid Tumor Patients During the COVID-19 Pandemic (2022 Edition)” (12).

### 2.3 COVID-19 diagnosis and typing

Patients who meet any of the following criteria were diagnosed with COVID-19: (a) nasopharyngeal swabs positive for SARS-CoV-2 nucleic acid; (b) rapid antigen test positive for SARS-CoV-2; (c) chest computed tomography (CT) findings consistent with COVID-19; (d) clinical diagnosis based on symptoms (no negative nucleic acid test results) and a history of close contact with confirmed COVID-19 patients within 14 days.

Disease severity was classified as asymptomatic, mild, medium, severe, and critical in accordance with the NCCN “Prevention and Treatment of Cancer-Related Infections (Version 3.2022)” guidelines (11). For symptomatic patients, the first onset of symptoms was considered the first day of infection. For asymptomatic patients, the date of the first positive nucleic acid or antigen test was considered the first day of infection. To ensure the safety of tumor patients during the COVID-19 pandemic, doctors collected information on the patients’ SARS-CoV-2 infection by telephone and through questionnaires and assessed their COVID-19 severity before antitumor treatment. The content of the questionnaire includes COVID-19 diagnostic method, symptoms, duration of symptoms, first day of infection and vaccination. For patients with positive results of SARS-CoV-2 nucleic acid or antigen test, we verified them in the medical record.

### 2.4 Treatment regimens

The neoadjuvant and adjuvant treatment regimens for breast cancer were formulated by referring to breast cancer clinical diagnosis and treatment guidelines issued by the NCCN and Chinese Society of Clinical Oncology (CSCO) (13, 14). Advanced stage rescue treatment plans were determined through the experience of the attending physician. The treatment plans included but were not limited to the TCbHP regimen (taxanes + carboplatin + trastuzumab + pertuzumab), TA regimen (taxanes + anthracyclines), HP regimen (trastuzumab + pertuzumab), AC-T regimen (anthracyclines + cyclophosphamide, sequential taxanes), TC+H regimen (taxanes + cyclophosphamide + trastuzumab), and NP regimen (navelbine + platinum). The treatment information of all patients was recorded in the patient’s medical records.

### 2.5 Grading and preventive management of treatment-related adverse reactions

The Common Terminology Criteria for Adverse Events (CTCAE) version 5.0 was used for grading adverse reactions after treatment. In our center, a full-time clinical pharmacist was responsible for following up on the adverse reactions of patients in each cycle and evaluating the adverse reactions. We used that data with the patients’ knowledge.

The following prophylactic treatments were administered for adverse reactions. (a) For nausea, a 5-HT3 receptor antagonist and dexamethasone were routinely given as prophylactic antiemetics. An NK-1 receptor antagonist was administered to patients with refractory vomiting. (b) For level I/II prevention of neutropenia, patients who were receiving taxane-containing drug regimens were administered prophylactic recombinant human granulocyte colony-stimulating factor (rhG-CSF) or polyethylene glycol rhG-CSF (PEG-rhG-CSF) 24 hours after chemotherapy, and the dosage was adjusted based on routine blood monitoring results (level I prevention). G-CSF was not administered to patients who were receiving low-risk chemotherapy regimens without taxane. If neutropenia occurred in the previous treatment cycle, G-CSF was used prophylactically in the next treatment cycle (level II prevention). (c) To manage cardiotoxicity related to trastuzumab/pertuzumab, cardiac function was monitored every 3 months during the use of targeted drugs, and subsequent use of targeted drugs was based on the monitoring results.

### 2.6 Statistical methods

R 4.1.2 software was used data management and statistical analyses. Measurement data are presented as the mean (standard deviation [SD]) for data that were normally distributed or median (interquartile range [IQR]) for data that were not normally distributed. Categorical data are presented as the frequency and percentage (%). Because this was a self-controlled study, the pai/red-sample χ^2^ test (McNemar’s test) was used to compare adverse reactions between periods. All tests were two-sided tests, and *p* < 0.05 was considered statistically significant.

## 3 Results

### 3.1 Clinicopathological data

The 109 breast cancer patients enrolled in the study were all women (**Table 1**) and were between 34 and 73 years of age (53.4 ± 10.3). Among all included patients, 15 (13.8%) were obese, 37 (33.9%) were overweight, and 52 (47.7%) had BMIs within the normal range. Most patients (66, 60.6%) had no comorbidity, in addition to breast cancer. The most prevalent comorbidities were hypertension (29, 26.6%), diabetes (20, 18.3%), liver (10, 9.2%), and heart disease (8, 7.3%).

**Table 1:** Descriptive statistics of the clinical features.

Characteristic	No. (%)
	Total (n=109)
Age, years [Mean (SD)]	53.4 (10.3)
Sex Female Male	109 (100) 0 (0)
BMI, kg/m^2^ [Mean (SD)] <18.5 ≥18.5, <24.0 ≥24.0, <28.0 ≥28.0	24.3 (3.4) 5 (4.6) 52 (47.7) 37 (33.9) 15 (13.8)
Comorbidities Type 2 Diabetes Mellitus Hypertension Lung disease Heart Disease Renal disease Liver disease Other primary cancer	20 (18.3) 29 (26.6) 0 (0) 8 (7.3) 0 (0) 10 (9.2) 4 (3.7)
Number of comorbidities 0 1 2 ≥3	66 (60.6) 23 (21.1) 12 (11.0) 8 (7.3)
Stage I II III IV	21 (19.3) 54 (49.5) 13 (11.9) 21 (19.3)
Tumor subtype HR+HER2- HR+HER2+ HR-HER2+ HR-HER2-	30 (27.5) 49 (45.0) 22 (20.2) 8 (7.3)
Histology Type Invasive ductal carcinoma Invasive lobular carcinoma Others	100 (91.7) 1 (0.9) 8 (7.3)
Grade I II III	7 (6.4) 52 (47.7) 50 (45.9)
Cancer treatment phase Neoadjuvant (%) Adjuvant (%) Salvage (%)	28 (25.7) 60 (55.0) 21 (19.3)
Cancer treatment regimen TCbHP THP TA TC AC T HP H Others	19 (17.4) 7 (6.4) 13 (11.9) 8 (7.3) 6 (5.5) 4 (3.7) 32 (29.4) 5 (4.6) 15 (13.8)
Hormone therapy Yes No	77 (70.6) 32 (29.4)
Delayed cancer treatment Yes No	89 (81.7) 20 (18.3)
Delay time, days [Media (IQR)]	18 (7-24)

(BMI, body mass index; HR, hormone receptor; HER2, human epidermal growth factor receptor 2; T, taxanes; Cb, carboplatin; H, trastuzumab; P, pertuzumab; A, anthracyclines; C, cyclophosphamide).

Preoperative biopsy and postoperative pathology showed that 79 patients (72.5%) had hormone receptor (HR) positive breast cancer, 71 patients (65.2%) had human epidermal growth factor receptor 2 (HER2) positive breast cancer, and 8 patients (7.3%) had triple-negative breast cancer (TNBC). The most common histology subtype (100, 91.7%) was invasive ductal carcinoma (IDC). Most patients (52, 47.7%) presented with grade II tumors, while 50 patients (45.9%) had grade III tumors, and 7 patients (6.4%) had grade I tumors.

Patients were divided into a neoadjuvant therapy group (25.7%), an adjuvant therapy group (55.0%), and an advanced rescue therapy group (19.3%) based on treatment phase. Most patients were hospitalized to receive taxane- or anthracycline-containing cytotoxicity treatment or anti-HER2-targeted therapy, and some patients (29.4%) received endocrine therapy concurrently. Among the patients enrolled in this study, 89 (81.7%) delayed antitumor treatment due to the recent COVID-19 wave, with an average delay of 16.4 days (SD 11.4).

### 3.2 Characteristics of COVID-19 infection in breast cancer patients

Among the 109 breast cancer patients with COVID-19 who received systemic therapy in our center, 99 (90.8%) had mild COVID-19. Although all patients were actively receiving breast cancer therapy that may lead to immunosuppression, no patients required hospitalization for severe or critical COVID-19 (**Table 2**). The dates of infection ranged from December 6, 2022, to December 27, 2022; during that period, Omicron strains were the predominant variants in Beijing. The most common COVID-19 symptoms were fever (n = 92, 84.4%), cough (n = 97, 83.5%), gastrointestinal symptoms (n = 26, 23.9%), sore throat (n = 39, 35.8%), and loss of taste and smell (n = 29, 26.6%); 15 (13.8%) patients still had sequelae of COVID-19 while receiving antitumor treatment, all of whom presented with mild dry cough, with no obvious aggravation after treatment. In addition, the vast majority of patients (n=86, 78.9%) had previously received COVID-19 vaccinations.

**Table 2:** Characteristics related to the COVID-19 exposure.

Characteristic	No. (%)
COVID-19 severity Mild Moderate Severe Critical	99 (90.8) 10 (9.2) 0 0
Symptoms and signs Fever Cough GI symptom Sore throat Loss of taste and smell	92 (84.4) 97 (83.5) 26 (23.9) 39 (35.8) 29 (26.6)
Duration of symptoms 1-7 days 7-14 days >14 days	34 (31.2) 43 (39.4) 32 (29.4)
COVID‐19 symptoms before cancer-directed therapy Asymptomatic Symptomatic	94 (86.2) 15 (13.8)
Time from COVID-19 diagnosis to cancer-directed therapy 14-21 days 21-28 days	45 (41.3) 64 (58.7)
Vaccination Yes No	86 (78.9) 23 (21.1)

### 3.3 Influence of COVID-19 infection on adverse reactions after anti-tumor therapy

In this study, breast cancer patients received antitumor therapy within 28 days after the diagnosis of COVID-19. The number of overall adverse events related to treatment (n = 87 [79.8%] *vs.* n = 91 [83.5%]; p = 0.42) and the number of serious adverse events of grade 3 or higher (n = 13 [11.9%] vs. n = 12 [11.0%]; p = 1.00) were similar between the observation period and the control period. The most common adverse events were peripheral neuropathy (n = 32 [29.4%]), pain (n = 29 [26.6%]), fatigue (n = 28 [25.7%]), nausea (n = 23 [21.1%]) and neutropenia (n = 19 [17.4%]), with no significant difference between periods (**Table 3**). The incidence of grade 3 and above adverse events was generally low. No cardiac adverse events were reported during the observation period, and no patients discontinued antitumor therapy due to adverse events.

**Table 3:** Results of self-controlled analysis on treatment-related AEs.

	All Grade ^a^			Grade ≥ 3		
	Infected (n=109)	Control (n=109)	P-value	Infected (n=109)	Control (n=109)	P-value
Gastrointestinal disorders, No. (%) Nausea Vomiting Diarrhea Constipation Loss of appetite	21 (19.3) 10 (9.2) 17 (15.6) 15 (13.8) 16 (14.7)	23 (21.1) 6 (28.6) 19 (17.4) 19 (17.4) 18 (16.5)	0.82 0.42 0.81 0.48 0.83	3 (2.8) 0 0 0 2 (1.8)	2 (1.8) 0 3 (2.8) 0 2 (1.8)	1.00 - 0.25 - 1.00
Hematologic disorders, No. (%) Neutropenia Anemia Leukopenia Thrombocytopenia	16 (14.7) 5 (4.6) 20 (18.3) 6 (5.5)	14 (12.8) 6 (5.5) 19 (17.4) 4 (3.7)	0.79 1.00 1.00 0.48	8 (7.3) 0 4 (3.7) 1 (<1%)	5 (4.6) 0 5 (4.6) 0	0.45 - 1.00 1.00
Others, No. (%)						
Pain	22 (20.2)	29 (26.6)	0.25	1 (<1%)	1 (<1%)	1.00
Rash	6 (5.5)	8 (7.3)	0.79	0	0	–
Neuropathy ^b^	29 (26.6)	32 (29.4)	0.73	2 (1.8)	1 (<1%)	1.00
Fatigue	23 (21.1)	28 (25.7)	0.44	0	0	–
Agrypnia	19 (17.4)	17 (15.6)	0.83	0	0	–
Raised aminotransferase	18 (16.5)	15 (13.8)	0.45	1 (<1%)	0	1.00
Total	87 (79.8)	91 (83.5)	0.42	13 (11.9)	12 (11.0)	1.00

a Patients could report more than one adverse event. Adverse events with an incidence of 5% or greater in either group are listed in this table. All adverse events were assessed under the National Cancer Institute Common Terminology Criteria for Adverse Events, version 5.0.

b Combined preferred term covering gait disturbance, hypoesthesia, muscular weakness, neuropathy peripheral, paresthesia, and peripheral sensory neuropathy.

"-" means N/A.

Stratified by age, treatment regimen and treatment stage, patients younger than 60 years old, in the neoadjuvant treatment stage, and receiving cytotoxic drugs had a higher overall adverse event rate; and stratified by COVID-19 factors, patients with COVID-19 sequelae during treatment, with a shorter interval between antitumor therapy and COVID-19, and without vaccination had a higher overall adverse event rate. However, in the abovementioned breast cancer patient population with a higher incidence of treatment-related adverse events, COVID-19 did not increase the occurrence of adverse events after antitumor therapy (**Table 4**).

**Table 4:** Subgroup analyses for the association between COVID-19 and treatment-related AEs.

	No. of patients	All Grade (No. (%))			Grade ≥ 3 (No. (%))		
		Infected	Control	P-value	Infected	Control	P-value
Age <60 ≥60	75 34	62 (82.7) 25 (73.5)	63 (84.0) 28 (82.4)	1.00 0.25	8 (10.7) 5 (14.7)	6 (8.0) 6 (17.6)	0.72 1.00
Cancer treatment phase Neoadjuvant therapy Adjuvant therapy Salvage therapy	28 60 21	27 (96.4) 45 (75.0) 15 (71.4)	28 (100) 47 (78.3) 16 (76.2)	1.00 0.72 1.00	8 (28.6) 5 (8.3) 0	6 (21.4) 5 (8.3) 1 (4.8)	0.72 1.00 1.00
Cancer treatment regimen Cytotoxic chemotherapy Non-cytotoxic treatment	65 44	62 (95.4) 25 (56.8)	63 (96.9) 28 (63.6)	1.00 0.55	13 (20.0) 0	10 (15.4) 2 (4.5)	0.58 0.48
Hormone therapy Yes No	32 77	20 (62.5) 67 (87.0)	23 (71.9) 68 (88.3)	0.45 1.00	1 (3.1) 12 (15.6)	2 (6.3) 10 (13.0)	1.00 0.77
Time since COVID-19 diagnosis to cancer-directed therapy 14-21 days 21-28 days	45 64	39 (86.7) 48 (75.0)	42 (93.3) 49 (76.6)	0.37 1.00	6 (13.3) 7 (10.9)	5 (11.1) 7 (10.9)	1.00 1.00
COVID‐19 symptoms before cancer-directed therapy Asymptomatic Symptomatic	94 15	74 (78.7) 13 (86.7)	77 (81.9) 14 (93.3)	0.58 1.00	12 (12.8) 1 (6.7)	11 (11.7) 1 (6.7)	1.00 1.00
Vaccination Yes No	86 23	68 (79.1) 19 (82.6)	69 (80.2) 22 (95.7)	1.00 0.25	10 (11.6) 3 (13.0)	11 (12.8) 1 (4.3)	1.00 0.62

## 4 Discussion

In this single-center retrospective study, with breast cancer treatment-related adverse reactions as the primary endpoint, the direct risk of breast cancer patients receiving antitumor therapy within 28 days after having COVID-19 was assessed. The COVID-19 pandemic has triggered delays in tumor screening, diagnosis and treatment (15, 16), and uncertainty about the safety of antitumor therapy after patients have COVID-19 may lead to further delays in antitumor treatment. Among solid tumors, breast cancer has the highest incidence among women, and the comprehensive treatment effects are good. However, delayed chemotherapy and targeted therapy may have substantial impacts on treatment efficacy. In this study, in patients at any stage of breast cancer treatment, whether they were undergoing chemotherapy, targeted therapy, or endocrine therapy, the incidence and severity of treatment-related adverse reactions did not significantly increase when receiving breast cancer treatment 2 to 4 weeks after having COVID-19, even in the group of patients with COVID-19 sequelae during antitumor therapy.

In the early stage of the COVID-19 outbreak, some studies found that SARS-CoV-2 nucleic acid clearance took longer in tumor patients than in the general population, with a median clearance time of 42 days (17). Considering the weakened immune function of tumor patients, previous studies have mostly focused on the risk of severe disease or death from COVID-19 in this patient group. Initial exploratory studies suggested that cancer patients who had recently received chemotherapy or surgery were at a higher risk of COVID-19-related death and that different types of antitumor treatments had different effects on the risk of severe COVID-19 (3, 18). Subsequently, a cohort study based on the COVID-19 and Cancer Consortium database (CCC19) showed that cancer patients had an increased risk of death due to COVID-19 but that the 30-day all-cause mortality was not significantly associated with recent cytotoxic systemic therapy and non-cytotoxic systemic therapy (19). Another prospective study also showed that compared with patients who did not receive antitumor therapy, patients who received immunotherapy, chemotherapy, endocrine therapy, and targeted therapy within 4 weeks of being diagnosed with COVID-19 did not have an increased COVID-19-related mortality rate (4). In our study, the patients with antitumor treatment did not experience severe COVID-19, and no exacerbation of symptoms was observed even in patients who were still symptomatic at the time of antitumor therapy. Previous studies showed that advanced age and comorbidities were significantly associated with hospitalization (20). In our study, the average age of patients is less than 60 years old, so there were fewer comorbidities. Moreover, breast cancer patients were more concerned about COVID-19 symptoms and received earlier symptomatic treatment, which maybe another reason of no severe disease. Actually, the Omicron strains that appear to carry less thrombotic risk compared to more virulent variants such as alpha or delta (21), were the predominant variants in Beijing during the infection period studied. The majority of patients (n=86, 78.9%) had previously received COVID-19 vaccinations and the possibility of weakened harmful effects of COVID-19 infection related antitumor therapy may also be considered as one of the reasons why no patients required hospitalization for severe or critical COVID-19 in our study. However, no data on the impact of vaccines on antitumor therapy after COVID-19 have been reported and further research on this issue is needed in the future.

Only few retrospective studies preceded ours in specifically evaluating the impact of having COVID-19 on tumor treatment-related adverse events. Di Lisi et al. (7) found an increased incidence of cancer treatment-related cardiac dysfunction (CTRCD) in cancer patients receiving anthracycline-containing drugs after the peak of the COVID-19 pandemic, a phenomenon that researchers believe may have been driven by failure to complete standard adverse cardiac event monitoring because of a decreased frequency of hospital visits during the pandemic. In our study, no cardiotoxicity events caused by anthracyclines, trastuzumab, or pertuzumab were observed, which may be related to the routine cardiac function monitoring in our center during the COVID-19 pandemic. Another study assessed organ function in relation to systemic melanoma therapy (e.g., immune-related adverse events, and cardiomyopathy caused by targeted drugs) and concluded that patients with melanoma on targeted or immune therapy did not have enhanced organ damage after COVID-19 (8). This is similar to our findings.

COVID-19 infection is thought to be associated with the excessive activation of immune responses. Cytokine storms accelerate the progression of COVID-19 and may induce acute respiratory distress syndrome (ARDS) (22). Patients with severe COVID-19 are characterized by a decrease in the number of total lymphocytes, CD8+ T cells, and monocytes and an increase in the expression levels of T-cell exhaustion markers such as PD-1 (23, 24). More than half of the patients in this study were treated with anti-HER2 targeted therapy drugs for breast cancer, i.e., trastuzumab and pertuzumab, both of which are human monoclonal antibodies. In addition to directly inhibiting HER2, their mechanism of action also involves antibody-dependent cell-mediated cytotoxicity (ADCC), and changes in the immune environment after having COVID-19 may have an impact on the effects of the above drugs (25). According to our data, there was no significant increase in the short-term toxic effects of targeted therapy during the COVID-19 pandemic; however, the impact of having COVID-19 and the resulting delay in treatment on the long-term efficacy of systemic breast cancer treatment requires long-term follow-up studies.

Most of the studies on SARS-CoV-2 infection in the patient population were done at the beginning of the pandemic in 2020. In the next three years, the emergence of the Alpha, Beta, Gamma, Delta, and Omicron variants has caused multiple waves of infection worldwide. The Omicron strains that emerged in late 2021 spread faster than previous strains but are associated with relatively mild clinical symptoms (26). An analysis of local infections in Beijing from November 14, 2022, to December 20, 2022, showed that more than 90% of the cases were caused by the Omicron variant subclade, of which the dominant strain BF.7 accounted for 75.7% and BA5.2 accounted for 16.3% (27). In view of the impact of variant strains on the course of emerging infectious diseases, this retrospective study is the first analysis of adverse effects of antitumor therapy after the epidemic wave involving the Omicron strains.

This study has the following limitations. First, this study was a single-center retrospective study, and the level of evidence is limited. Second, the aim of this study was to quickly provide data on the safety of antitumor treatment after having COVID-19, and thus, the sample size is relatively insufficient, which may carry a risk of type II error. Third, this study only compared the incidence of adverse reactions in the short term after treatment. The toxicity of some chemotherapeutic drugs (such as anthracyclines) is related to the cumulative dose; however, the control period in this study was the period before the COVID-19 pandemic, when the cumulative dose of chemotherapy drugs was relatively low. Fourth, the criteria for diagnosis of COVID-19 includes non-stringent criteria based on clinical features and the infection maybe overestimated. Fifth, during the COVID-19 pandemic, patients may have been more concerned about symptoms and increased their use of preventive drugs (such as G-CSF), thus masking the high risk of tumor treatment-related adverse reactions due to COVID-19.

## 5 Conclusion

Restarting antitumor therapy 2-4 weeks after having mild or moderate COVID-19 is a relatively safe strategy for breast cancer patients that does not increase the risk of treatment-related adverse events and reduces delays in antitumor therapy. Until more research results are reported, the antitumor treatment for patients after having COVID-19 must be conducted with caution, and it is necessary to strengthen adverse reaction monitoring and prevention strategies during treatment.

## Data availability statement

The raw data supporting the conclusions of this article will be made available by the authors, without undue reservation.

## Ethics statement

The studies involving humans were approved by the Biomedical Research Ethics Committee of Peking University First Hospital (No. 2023 133-001). The studies were conducted in accordance with the local legislation and institutional requirements. The participants provided their written informed consent to participate in this study.

## Author contributions

QL, LX, and JY contributed to conception and design of the study. MD, YC, and HX organized the database. MD performed the statistical analysis. MD wrote the first draft of the manuscript. QL, YC, and LX wrote sections of the manuscript. All authors contributed to manuscript revision, read, and approved the submitted version.
